# Special Issue for Purinergic Receptors, Particularly P2X_7_ Receptor, in the Eye

**DOI:** 10.3390/vision2030030

**Published:** 2018-07-24

**Authors:** Tetsuya Sugiyama

**Affiliations:** 1 Nakano Eye Clinic of Kyoto Medical Co-operative, Kyoto 604-8404, Japan; tsugiyama7@kyo-con.or.jp; Tel.: +81-75-801-4151; 2 Department of Ophthalmology, Osaka Medical College, Takatsuki, Osaka 569-8686, Japan; 3 Department of Ophthalmology, Toho University Sakura Medical Center, Sakura, Chiba 285-8741, Japan

Purinergic receptors, also known as purinoceptors, are a family of plasma membrane molecules found in almost all mammalian tissues [1]. In the field of purinergic signaling, these receptors play a role in many physiological functions, such as learning, memory, locomotion, and sleep [2]. Specifically, they are involved in several cellular functions including proliferation and migration of neural stem cells, vascular reactivity, apoptosis, and cytokine secretion [2,3]. The term ‘purinergic receptor’ was originally introduced to describe specific classes of membrane receptors that mediate relaxation of gastrointestinal smooth muscle as a response to the release of ATP—adenosine triphosphate (P2 receptors) or adenosine (P1 receptors). P2 receptors have been further divided into two subclasses: P2X and P2Y [4]. P2X receptors are a family of ligand-gated ion channels that allow the passage of ions across cell membranes. P2Y receptors are a family of G-protein-coupled receptors that initiate an intracellular chain of reactions.

Some purinergic receptors have been found to play important roles in ocular tissues including the lacrimal gland, the cornea, the trabecular meshwork, the lens, and the retina [5]. For example, the P2X_7_ receptor possesses unique features that are likely to be of both physiological and pathophysiological significance in the retina. Most importantly, not only does the initial activation of these receptors result in the opening of a non-selective plasma membrane channel, but in many types of cells, sustained activation causes the formation of trans-membrane pores that are permeable to hydrophilic molecules of up to 900 Da [6,7].

Expression of the P2X_7_ receptor has been demonstrated in most types of retinal cells; these include neurons, such as the retinal ganglion cells [8,9,10], as well as glia [11,12] and vascular cells [13]. For instance, stimulation of P2X_7_ receptor has been reported to be involved in neuronal and microvascular cell death under pathogenic conditions like ischemia [14], glaucoma [15,16,17,18], and diabetic retinopathy [19,20].

The current Special Issue was open to submissions of unpublished experimental articles and review papers on the fundamental basis of the physiological and pathological roles of purinergic receptors, and purinergic receptors as a potential therapeutic target for ocular diseases. Consequently, three suggestive review papers and one valuable experimental article, all of which help us understand the involvement of P2X_7_ receptor in ocular diseases, have been published. These articles are briefly summarized below.

Although the P2X_7_ receptor has been identified in the retinal pigment epithelium (RPE) and different retinal layers, its biological and pathological functions, as well as its downstream signaling pathways in the RPE and retina, are not yet fully understood. Yang’s review paper [21] mainly focuses on recent findings of in vitro and in vivo evidence for the role of the P2X_7_ receptor in the RPE and in age-related macular degeneration (AMD).

Dutot et al. [22] summarize the role of the P2X_7_ receptor in exogenous and endogenous ocular stresses as well as in ocular diseases including AMD, diabetic retinopathy, and glaucoma. Treatments to inhibit P2X_7_ receptor activation are proposed, using either eye-drops or food supplements.

Subauste presents a review paper [23] on the implication of CD40, a novel inducer of purinergic signaling, in the pathogenesis of diabetic retinopathy. CD40 is an upstream inducer of a broad range of inflammatory responses in the diabetic retina and is required for death of retinal capillary cells. Recent studies have uncovered CD40 as a novel inducer of purinergic signaling and identified the CD40-ATP-P2X_7_ pathway as playing a key role in the induction of inflammation in the diabetic retina and the programmed cell death of retinal endothelial cells.

P2X_7_ receptor/channels in the retinal microvasculature not only regulate vasomotor activity but can also trigger death of capillary cells. While it is known that this purinergic vasotoxicity is dependent on the transmembrane pores that form during P2X_7_ activation, events linking pore formation with cell death remain uncertain. Shibata et al. [24] reveal that the previously unappreciated pore/oxidant/KATP channel/Ca^2+^ pathway accounts for 75% of the capillary cell death triggered by sustained P2X_7_ receptor/channel activation. Elucidation of this pathway is of potential therapeutic importance since purinergic vasotoxicity may play a role in sight-threatening disorders such as diabetic retinopathy.

I believe these papers could lead to a new strategy for treating some ocular diseases. I close this editorial, expecting many advancements in this field in the near future.
